# High incidence of atrial fibrillation after successful catheter ablation of atrioventricular nodal reentrant tachycardia: a 15.5-year follow-up

**DOI:** 10.1038/s41598-019-47980-1

**Published:** 2019-08-13

**Authors:** M. K. Frey, B. Richter, M. Gwechenberger, M. Marx, T. Pezawas, L. Schrutka, H. Gössinger

**Affiliations:** 1 Department of Cardiology, Medical University Vienna, Waehringer Guertel 18-20, 1090 Vienna, Austria; 2 Department of Pediatric Cardiology, Medical University Vienna, Waehringer Guertel 18-20, 1090 Vienna, Austria

**Keywords:** Interventional cardiology, Atrial fibrillation

## Abstract

Atrioventricular nodal reentrant tachycardia (AVNRT) is the most common type of supraventricular tachycardia. Slow pathway (SP) ablation is the treatment of choice with a high acute success rate and a negligible periprocedural risk. However, long-term outcome data are scarce. The aim of this study was to assess long-term outcome and arrhythmia free survival after SP ablation. In this study, 534 consecutive patients with AVNRT, who underwent SP ablation between 1994 and 1999 were included. During a mean follow-up of 15.5 years, 101 (18.9%) patients died unrelated to the procedure or any arrhythmia. Data were collected by completing a questionnaire and/or contacting patients. Clinical information was obtained from 329 patients (61.6%) who constitute the final study cohort. During the electrophysiological study, sustained 1:1 slow AV nodal pathway conduction was eliminated in all patients. Recurrence of AVNRT was documented in 9 patients (2.7%), among those 7 patients underwent a successful repeat ablation procedure. New-onset atrial fibrillation (AF) was documented in 39 patients (11.9%) during follow-up. Pre-existing arterial hypertension (odds ratio 2.61, 95% CI 1.14–5.97, p = 0.023), age (odds ratio 1.05, 95% CI 1.02–1.09, p = 0.003) and the postinterventional AH interval (odds ratio 1.02, 95% CI 1.00–1.04, p = 0.038) predicted the occurrence of AF. The present long-term observational study after successful SP ablation of AVNRT confirms its clinical value reflected by low recurrence and complication rates. The unexpectedly high incidence of new-onset AF (11.9%) may impact long-term follow-up and requires further clinical attention.

## Introduction

Atrioventricular nodal reentrant tachycardia (AVNRT) is the most common type of paroxysmal supraventricular tachycardia^1^. Usually young adults without structural or ischemic heart disease are affected, and more than 60% of cases are observed in women^2^. Catheter ablation of the slow pathway (SP) has evolved as the treatment of choice over long-term pharmacologic therapy for symptomatic AVNRT^3^. Hereby, the preferred technique is SP ablation using radiofrequency (RF)^4,5^. Risk of acute complications like complete AV block is very low^6^ and recurrence rates are approximately 3% to 7%^7,8^. In contrast, drug efficacy is in the range of 30 to 50%^9^. In consequence, catheter ablation is the treatment of first choice for symptomatic AVNRT and treated patients are normally discharged without regular follow-up or specific recommendations.

However, it is known that AVNRT can coexist with atrial fibrillation (AF), the most frequent sustained arrhythmia which is associated with an increase in all-cause mortality and morbidity^10^. Ozcan *et al*. report that the presence of any form of supraventricular arrhythmia is associated with an increased risk for the development of AF^11^. In this study, 9.7% of patients with AVNRT had concomitant AF. In another study, successful SP ablation markedly reduced the AF recurrence rate in symptomatic patients with AVNRT and pre-existent AF^12^. In patients with manifest or concealed accessory pathways and documented AF, older age was a significant independent predictor for the recurrence of AF after RF ablation of the accessory pathway^13^. Possible mechanisms of AF initiation in these patients include enhanced atrial vulnerability and degeneration of AVNRT into AF.

Until now, data about long-term outcome after AVNRT ablation are sparse. Most studies report a follow-up period of 2 to 5 years^12,14^. To the best of our knowledge, the longest mean follow-up after SP ablation is 9 years, however the study size of 47 patients was rather small^15^. In this study, no recurrences occurred more than 3 years after ablation. Making a statement about long-term AVNRT recurrence rates and incidence of other arrhythmia after SP ablation requires a longer observation period. This retrospective observational study was aimed to assess long-term outcome, in particular the survival free of any arrhythmia and the incidence of PM implantation after SP ablation.

## Methods

### Study design

Between January 1993 and December 1999, 534 consecutive patients who underwent RF catheter ablation of AVNRT at our institution were included in this retrospective long-term follow-up study. The study was performed in accordance with the Declaration of Helsinki and approved by the local Ethics Committee of the Medical University of Vienna. The participants were required to sign an informed consent. All patients had frequent symptomatic episodes of AVNRT before the ablation procedure. To obtain the follow-up information, patients were asked to complete a questionnaire and/or contacted by telephone. Furthermore, medical files were screened for documented recurrence, new-onset arrhythmias and repeat electrophysiological studies. Additional information regarding arrhythmias was obtained from the referring cardiologists who were also asked to send available electrocardiograms of these arrhythmias. Time and causes of death were obtained from a national survey (Statistik Austria, Vienna, Austria) and from hospital records.

### Electrophysiologic study protocol

Atrioventricular nodal re-entrant tachycardia was diagnosed on the basis of standard criteria^9^. Antiarrhythmic drugs had been discontinued at least five half-lives before the study. Conscious sedation using fentanyl and midazolam was used. Under local anaesthesia with lidocaine four multielectrode catheters (Cordis Webster, Miami, FL, USA) were inserted percutaneously into the heart via both femoral veins. Two quadripolar catheters were placed in the high right atrium and the right ventricular outflow tract. A hexapolar catheter was positioned at the AV junction to record His-bundle activation, and a 4- mm-tip catheter was used for mapping and ablation of the slow pathway along the tricuspid annulus. All recordings were stored in a digital system (Bard Electrophysiology. Tewksbury. MA, USA). Measurements were made from the computer screen at a sweep speed of 2(K) mm/sec. The study protocol included programmed atrial stimulation with single premature extrastimuli, incremental atrial pacing, and incremental ventricular pacing.

### RF catheter ablation

After initiation of the tachycardia by atrial pacing and confirmation of the diagnosis of AVNRT, catheter ablation of the slow pathway was performed with a commercially available RF generator (Radionics). RF pulses were delivered at 30 to 35 Watt. The technique used was a combination of the anatomic and electrogram mapping approaches as described in detail previously^16^. The end point of the ablation procedure was defined as the inability to induce AVNRT. This was achieved either by complete elimination of slow pathway conduction, as evaluated by both extrastimulus testing and incremental pacing, or by impairment of the slow pathway to the extent that it could not sustain 1:1 AV conduction.

### Statistical analysis

Data are expressed as mean ± standard deviation (SD) if normally distributed, or otherwise by median (interquartile range). Univariate and multivariate logistic regression analysis was used to identify variables predictive of new-onset AF. In the final mulivariate analysis all variables significant at univariate analysis were entered. All statistical analyses were performed using SPSS for Windows, version 20 (SPSS Inc, Chicago, IL). A two tailed P value < 0.05 was considered significant.

## Results

### Patient characteristics

During a mean follow-up of 15.5 +/− 2.3 years, 101 (18.9%) patients died unrelated to the procedure. Causes of death in our patient population were neoplasia (n = 29; 28.7%), coronary artery disease (n = 25; 24.8%), stroke (n = 6; 5.9%), respiratory diseases (n = 6; 5.9%), heart failure (n = 5; 5.0%), ventricular arrhythmias (n = 4; 4.0%), trauma (n = 3; 3.0%), renal failure (n = 3; 3.0%), peripheral artery disease (n = 2; 2.0%), aortic stenosis (n = 2; 2.0%), pulseless electrical activity (n = 1; 0.3%), miscellaneous (n = 12; 11.9%) or unknown (n = 4; 4.0%). One hundred and four patients (19.5%) were lost to clinical follow-up. We could obtain information from 329 patients (61.6%), who constitute the final study population. Demographic and baseline characteristics are shown in Table 1.

**Table 1:** Baseline characteristics of the study population *n* = 329.

Female, n (%)	238 (72.3)
Age (years)	46.5 (6.3–78.2)
BMI (kg/m²)	25.3 ± 4.5
Episodes per month	5.1 ± 1.4
Arterial hypertension, n (%)	71 (21.5)
CAD, n (%)	6 (1.8)
Diabetes, n (%)	7 (2.1)
Structural heart disease, n (%)	18 (5.5)
Previous pharmacological therapy, n (%)	
Beta blocker	53 (16.1)
ACEI/ARB	28 (8.5)
CCB	14 (4.3)
Diuretics	3 (0.9)
Amiodarone	0
Preexistent AF, n (%)	7 (2.1)
Preexistent atrial flutter, n (%)	1 (0.3)
Preexistent atrial tachycardia, n (%)	8 (2.4)
Preexistent LBBB, n (%)	8 (2.4)
Preexistent RBBB, n (%)	11 (3.3)
Preexistent SN dysfunction, n (%)	2 (0.6)
Nicotine abuse, n (%)	39 (11.8)
Systolic LV dysfunction, n (%)	4 (1.2)
Diastolic LV dysfunction, n (%)	61 (18.5)
LA enlargement, n (%)	59 (17.9)
RA enlargement, n (%)	39 (11.8)
LV hypertrophy, n (%)	25 (7.6)
Mitral regurgitation, n (%)	45 (13.6)
Tricuspid regurgitation, n (%)	37 (11.2)

Categorical variables are expressed as frequencies (n) and percentages (%). Skewed variables are presented as median (interquartile range). Abbreviations: ACEI angiotensin converting enzyme inhibitor, AF atrial fibrillation, ARB angiotensin receptor blocker, BMI body mass index, CAD Coronary artery disease, CCB calcium channel blocker, LA left atrium, LBBB left bundle branch block, LV left ventricle, RA right atrium, RBBB right bundle branch block, SN sinus node.

### Procedural characteristics and follow-up

Mean AVNRT cycle length was 363 ± 68 ms. ‘Slow –fast’ was the most common AVNRT type (96%). Atypical AVNRT was detected in 3.9% (slow-slow 2.4%, fast-slow 1.5%). The mean duration of RF application was 188 ± 129 seconds. Sustained 1:1 slow AV nodal pathway conduction was eliminated in all patients. An AH jump was still present after ablation in 117 patients (35.6%). AF was induced by incremental pacing during the electrophysiological study in 33.3% of all procedures, atrial flutter in 8.5% and focal atrial tachycardia in 7.3%. In 3.3% of patients, ablation of a second arrhythmia was performed at the same procedure (Table 2). There was no procedure-related AV-block requiring immediate pacemaker implantation. However, intermittent AV-block occurred in 4 patients (1.2%), of whom 1 patient presented with preexistant prolonged AH-interval in the setting of concomitant ventricular septal defect. In another patient, AV conduction returned to baseline values already at the end of the procedure. In the third patient there was delayed AV-nodal conduction in the post-ablation period. A repeat ablation due to recurrent AVNRT 3.8 years later led to persistent symptomatic second-degree AV block necessitating PM implantation. The forth patient with periprocedural AV-block was discharged with intermittent second-degree AV-blockade but did not require pacemaker implantation during follow-up. Prolongation of the AH interval during the intervention was observed in 143 cases (43.5%; mean prolongation 9.2 ± 7.4 ms). Significant prolongation (more than 30 ms) occurred in 1 patient. This patient had an uneventful follow-up. Procedure related complications comprised pseudoaneurysm of the femoral artery in 1 patient (0.3%) and minimal pericardial effusion not requiring paracentesis in 3 patients (0.9%).

**Table 2:** Procedural Characteristics.

AVNRT cycle length	363 ± 68 ms
typical AVNRT (slow/fast)	316 (96%)
atypical AVNRT	13 (3.9%)
slow/slow	8 (2.4%)
fast/slow	5 (1.5%)
RF duration	188 ± 129 s
Simultaneous ablation of	
Atrial flutter	1 (0.3%)
Atrial tachycardia	5 (1.5%)
RVOT PVC	2 (0.6%)
WPW	2 (0.6%)
Coumel tachycardia	1 (0.3%)
Inducible AF	110 (33.3%)
Inducible atrial flutter	28 (8.5%)
Inducible atrial tachycardia	24 (7.3%)
Complications	
AV blockade	4 (1.2%)
AV blockade requiring PM implantation	1 (0.3%)
PSA	1 (0.3%)
Pericardial effusion	3 (0.9%)

Categorical variables are expressed as frequencies (n) and percentages (%). Abbreviations: AF atrial fibrillation, AV atrioventricular, AVNRT atrioventricular nodal reentrant tachycardia, PM pacemaker, PSA pseudoaneurysm of femoral artery, RF radiofrequency, RVOT PVC premature ventricular contractions arising from the right ventricular outflow tract, WPW Wolff-Parkinson-White.

Acute success was obtained in all patients. Recurrence of AVNRT was documented in 9 patients (2.7%) 11 months to 10 years after ablation. Among those, 7 patients underwent a successful second ablation procedure. Complete elimination of slow AV nodal pathway conduction or the presence of single atrial beats conducted on the slow pathway had no effect on AVNRT recurrence rate (2.8% versus 2.6%). Patients presenting with late recurrences (more than 3 years after first ablation) did not differ from patients with early recurrences regarding baseline characteristics, procedural detail or outcome (Supplemental Table S1).

During follow-up, 39 patients (11.9%) developed AF, 1 patient (0.3%) atrial flutter and 1 patient (0.3%) atrial tachycardia (Table 3). Among 7 patients with known AF before SP-ablation, only 3 patients reported AF at follow-up (42.9%). Of 39 patients with AF at follow-up, 3 patients (7.7%) underwent successful pulmonary vein isolation later on. Here again, complete slow pathway elimination or the presence of residual AH jumps after ablation had no influence on rate of new-onset atrial fibrillation (11.8% versus 12.0%).

**Table 3:** Outcome.

Acute success	329 (100%)
Recurrence documented	9 (2.7%)
Repeated ablation	7 (2.1%)
New arrhythmia	
AF	39 (11.9%)
Atrial flutter	1 (0.3%)
Atrial tachycardia	1 (0.3%)
PM implantation	11 (3.3%)
AV conduction delay	2 (0.6%)

Categorical variables are expressed as frequencies (n) and percentages (%). Abbreviations: AF atrial fibrillation, AV atrioventricular, PM pacemaker.

Eleven patients (3.3%) underwent pacemaker implantation 8.6 ± 3.5 years after ablation due to symptomatic sick-sinus (n = 5), second degree AV-block (n = 5) and due to iatrogenic AV-block during valve surgery (n = 1). Only 1 of those patients receiving a pacemaker during the follow-up period had procedure-related AV nodal impairment with intermittent AV-blockade during SP ablation. This patient also underwent a repeated ablation because of recurrence of AVNRT and finally received a pacemaker due to second-degree AV-block 6 months after the second ablation procedure. Among the remaining 4 patients with pacemaker implantation due to second degree AV-block during follow-up, no particularities could be found regarding baseline characteristics or procedural details (Supplemental Table S2).

During the ablation procedure, 2 patients were diagnosed with additional pre-existing sick sinus syndrome (SSS) and 2 patients with pre-existing AV-nodal conduction impairment. All 4 patients did not require pacemaker implantation during follow-up.

### Predictors of new onset AF

Pre-existing arterial hypertension (odds ratio [OR] 2.61, 95% CI 1.14–5.97, p < 0.03), age (OR 1.05, 95% CI 1.02–1.09, p < 0.005) and the postinterventional AH interval (OR 1.02, 95% CI 1.01–1.04, p < 0.05) predicted the occurrence of AF, whereas the induction of AF by incremental pacing during the electrophysiological study was of no predictive value (OR 1.66, 95% CI 0.84–3.29, p = 0.14) (Table 4).

**Table 4:** Predictors of new-onset atrial fibrillation.

Variable	p-value	OR	95% CI
**Univariate predictors**			
Sex	0.344	0.71	0.3–1.4
Arterial hypertension	**0.000**	4.85	2.4–9.7
Diabetes	**0.024**	5.87	1.3–27.3
CAD	0.585	1.86	0.2–7.0
Structural heart disease	0.871	1.14	0.2–5.2
Systolic dysfunction	0.598	1.41	0.4–5.1
Diastolic dysfunction	0.097	1.91	0.9–4.1
LV hypertrophy	0.184	1.40	0.9–2.3
RF duration	0.976	1.00	1.0–1.0
Inducible AF	0.142	1.66	0.8–3.3
Inducible atrial flutter	0.094	2.30	0.9–6.1
Inducible atrial tachycardia	0.933	1.06	0.3–3.7
LA enlargement	**0.003**	2.93	1.5–5.9
RA enlargement	**0.001**	3.46	1.7–7.2
BMI	**0.026**	1.08	1.0–1.2
Age	**0.000**	1.10	1.0–1.1
AH interval before ablation	0.179	1.01	1.0–1.0
AH interval after ablation	**0.003**	1.03	1.0–1.1
AH interval difference	0.675	1.00	1.0–1.0
**Multivariate predictors**			
Arterial hypertension	**0.023**	2.61	1.1–6.0
Diabetes	0.083	4.90	0.8–28.2
LA enlargement	0.868	0.90	0.3–3.3
RA enlargement	0.487	1.59	0.4–5.8
BMI	0.696	0.98	0.9–1.1
Age	**0.003**	1.05	1.0–1.1
AH after ablation	**0.038**	1.02	1.0–1.0

Results of the binary logistic regression. Abbreviations: AF atrial fibrillation, BMI body mass index, CAD Coronary artery disease, CI confidence interval, LA left atrium, LV left ventricle, OR odds ratio, RA right atrium, RF radiofrequency.

## Discussion

The present study is the largest long-time follow-up study after SP ablation for symptomatic AVNRT, which revealed two main findings: firstly, SP ablation has a high long-term success rate and is associated with a negligible long-term pacemaker implantation rate. Secondly, the rate of new-onset AF was 11.9%, which is much higher than could be expected from general population-based data.

Our data show that extension of follow-up to a 15 year-period is still associated with a low recurrence rate of AVNRT (2.7%). Of note, recurrences occur up to 10 years after index ablation. This is in contrast to a long-time follow-up study after AVNRT by Kimman *et al*., where recurrences were not observed more than three years after ablation^15^. In our study, a repeat ablation performed in 2.1% of patients one to ten years after the initial procedure rendered all patients asymptomatic. Altough patient numbers were small, we were not able to detect differences in baseline characteristics or procedural details between patients with early and late recurrences (more than three years). As shown previously, periprocedural risk is negligible. In our registry, only 2.4% of patients suffered an in-hospital complication comprising intermittent AV-blockade (n = 4), pseudoaneurysm (n = 1) and pericardial effusion (n = 3) (Table 2). With regard to acute procedural success rates, recurrence rate and rate of complications our data favourably compare to previous results^17^.

Based on the results of our study we can reassure that the rate of pacemaker implantation during long-term follow-up is very low (3.3%) and usually attributed to conditions unrelated to the procedure such as sick-sinus-syndrome. Notably, procedure related AH prolongation does not play a role in the prediction of future PM implantation because only 2 out of 143 patients with AH prolongation (8 and 15 ms) required a PM implantation later on, whereas AH interval was unchanged or even shorter post ablation in the other 9 patients receiving a PM.

In this observational study, the patient population is definitely older (median age at ablation 46.5 years) than in newer reports on AVNRT ablation, a circumstance which may have implications for the relatively high mortality rate during long term follow-up. Explanation for the rather elderly patient population was a tailback of symptomatic AVNRT patients eagerly awaiting the introduction of this curative treatment modality in the early nineties. This explains a mean duration of symptoms of 12.9 years prior to ablation in our registry. Causes of death are comparable with the general population as reflected by a cardiovascular mortality rate of 43.6% and neoplasia associated mortality of 28.7%.

**Figure 1:**
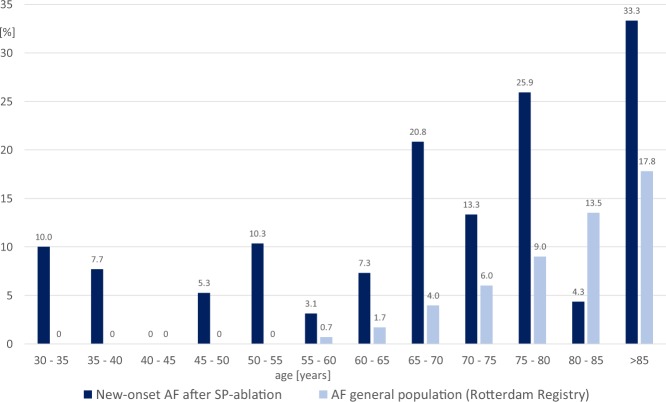
Incidence of new-onset atrial fibrillation after SP-ablation for AVNRT. AF atrial fibrillation, SP slow pathway.

An unexpected observation was that 11.9% of patients developed new-onset AF after successful SP ablation. Including also patients with pre-existent AF, the prevalence of AF after SP ablation in our study is even 12.8%. Comparing this number with the prevalence of AF in the common population, the risk to develop AF seems to be much higher in patients with AVNRT than in the general population. In a large prospective cohort study about the prevalence of AF in Europe, overall prevalence in the general population was 5.5%^18^. In patients aged 60 to 65 years, the prevalence of AF in this large registry including 6808 patients was 1.7%, whereas in our study 7.3% of patients in this age group suffered from AF (Fig. 1). Binary logistic regression revealed that arterial hypertension, age and postinterventional AH interval are associated with a higher risk to develop future AF (Table 4).

There are several possible mechanisms which could predispose patients with AVNRT to develop AF.

Ablation for any kind of arrhythmia *per se* could trigger AF by creating myocardial damage and a substrate for potential further arrhythmias. Haissaguere reported that persistence of atrial vulnerability as reflected by the induction of AF by atrial extrastimuli after catheter ablation of overt accessory pathways was the only factor associated with recurrence of AF in this population^19^. However, in our registry only 3 out of 7 patients (42.9%) with known pre-existing AF also reported AF at follow-up. This is in line with a previous study, where patients with AVNRT and concomitant AF undergoing SP-ablation were more likely to be AF free without taking any antiarrhythmic medication than a comparable control group without SP-ablation^12^. This highlights the role of AVNRT as trigger arrhythmia for AF in a selected subgroup of patients. In the absence of AVNRT, as shown in a few patients who underwent successful catheter ablation of AF, exclusive pulmonary vein isolation proved to be clinical effective.

Secondly, AVNRT and AF might share similar risk factors and therefore the rate of AF in our registry could be higher than in the general population. This hypothesis can also be disproved because large registries have shown that arterial hypertension in AVNRT patients is as frequent as in the general population. In our population 21.5% presented with pre-existent arterial hypertension, which is almost exactly the same rate as in the general population presented in the Rotterdam Registry, there 21.4% had arterial hypertension at baseline. Also other known risk factors for AF like coronary heart disease, cardiomyopathy or diabetes were found in a similar prevalence as in the general population.

Finally, genetic polymorphisms could predispose patients to both arrhythmia, AF and AVNRT. This concept has also been discussed in patients with Wolff-Parkinson-White (WPW) syndrome, where recurrent paroxysmal AF has been reported to occur in up to 15–20% of patients^20,21^. Similar to patients with WPW, also in AVNRT molecular mechanisms may be responsible for a higher rate of AF.

This study is a retrospective observational study that represents a single center experience and should be interpreted in the context of the following limitations:

Firstly, follow-up of our patients was not conducted systematically and repeat Holter ECG was not attained. First diagnosis of any new arrhythmia was made by general practitioners or referring hospitals and diagnosis was then confirmed by experienced cardiologists at our institution. We therefore cannot exclude that even more patients with previous SP ablation suffer from AF, especially if these patients experienced asymptomatic AF or were not followed by a physician at regular intervals. Secondly, patients in our registry are definitely older than in recent reports of AVNRT ablation which could have an impact on AF occurrence. However, AF was documented at an age as early as 33 years in one patient and, as displayed in Fig. 1, the increased occurrence of AF can be observed throughout the entire span of lifetime. Third, the response rate of 62% to our survey was only moderate which may have led to bias.

In conclusion, the present study which is the largest long-term follow-up study after ablation for AVNRT confirms that SP-ablation has a high success rate also more than fifteen years after ablation. The unexpectedly high incidence of new-onset AF may impact long-term follow-up and requires further clinical attention.

## Supplementary information

Supplemental Table S1 and S2

Supplemental Dataset 1

## Data Availability

The datasets generated during and/or analysed during the current study are available from the corresponding author on reasonable request.
