# Supplementary material for: High incidence of atrial fibrillation after successful catheter ablation of atrioventricular nodal reentrant tachycardia: a 15.5-year follow-up
Source: Sci Rep. 2019 Aug 13;9:11784. doi: 10.1038/s41598-019-47980-1 (PMC6692351; doi:10.1038/s41598-019-47980-1)
Supplement: Supplementary file 1 — Supplemental Table S1 and S2 [file 41598_2019_47980_MOESM1_ESM.docx]

High incidence of atrial fibrillation after successful catheter ablation of atrioventricular nodal reentrant tachycardia: a 15.5-year follow-up

MK Frey, MD^1.*^, B Richter, MD^1^, M Gwechenberger, MD^1^, M Marx, MD^2^, T Pezawas, MD^1^, L Schrutka, MD^1^, H Gössinger, MD^1^

^1^Department of Cardiology, Medical University Vienna, Waehringer Guertel 18-20, 1090 Vienna, Austria; ^2^Department of Pediatric Cardiology, Medical University Vienna, Waehringer Guertel 18-20, 1090 Vienna, Austria

*[maria.frey@muv.ac.at](mailto:maria.frey@muv.ac.at)

**Supplemental Table S1** Baseline characteristics, procedural details and outcome of patients with early recurrence (under 3 years) and late recurrence (more than 3 years) of AVNRT

|  | Early recurrence, n=5 | Late recurrence, n=4 |
| --- | --- | --- |
| Time until recurrence, years (range) | 1 (0.7-2.1) | 10 (9.3-11.3) |
| Age at first ablation, years (range) | 48 (13.4-53.4) | 31 (22.8-58) |
| Hypertension at baseline, n (%) | 2 (40) | 1 (25) |
| Diabetes at baseline, n (%) | 0 | 1 (25) |
| Procedural details, n (%) |  |  |
| inducible atrial fibrillation | 3 (60) | 1 (25) |
| inducible atrial tachycardia | 0 | 1 (25) |
| intermittant AV-blockade | 1 (20) | 0 |
| Drugs during follow-up, n (%) |  |  |
| Betablocker | 2 (40) | 2 (50) |
| Amiodarone | 1 (20) | 0 |
| Repeated ablation, n (%) | 3 (60) | 3 (75) |
| Outcome |  |  |
| pacemaker | 1 (20) | 0 |
| atrial fibrillation | 0 | 0 |

Categorical variables are expressed as frequencies (n) and percentages (%). Skewed variables are presented as median (interquartile range).

**Supplemental Table S2** Baseline characteristics, procedural details and outcome of patients requiring pacemaker implantation for AV block during follow-up (n=5)

| Baseline characteristics, n (%) |  |
| --- | --- |
| Preexistant AV blockade | 1 (20) |
| Atrial tachycardia | 1 (20) |
| Hypertension | 2 (40) |
| Hyperlipidaemia | 2 (40) |
| Procedural details, n (%) |  |
| inducible atrial fibrillation | 1 (20) |
| inducible atrial tachycardia | 1 (20) |
| intermittant AV-blockade | 1 (20) |
| Drugs during follow-up, n (%) |  |
| Betablocker | 0 |
| Calcium channel blocker | 0 |
| Amiodarone | 0 |
| Repeated ablation, n (%) | 1 (20) |
