# Supplementary material for: High incidence of atrial fibrillation after successful catheter ablation of atrioventricular nodal reentrant tachycardia: a 15.5-year follow-up
Source: Sci Rep. 2019 Aug 13;9:11784. doi: 10.1038/s41598-019-47980-1 (PMC6692351; doi:10.1038/s41598-019-47980-1)
Supplement: Supplementary file 2 — Supplemental Dataset 1 [file 41598_2019_47980_MOESM2_ESM.docx]

High incidence of atrial fibrillation after successful catheter ablation of atrioventricular nodal reentrant tachycardia: a 15.5-year follow-up

MK Frey, MD^1.*^, B Richter, MD^1^, M Gwechenberger, MD^1^, M Marx, MD^2^, T Pezawas, MD^1^, L Schrutka, MD^1^, H Gössinger, MD^1^

^1^Department of Cardiology, Medical University Vienna, Waehringer Guertel 18-20, 1090 Vienna, Austria; ^2^Department of Pediatric Cardiology, Medical University Vienna, Waehringer Guertel 18-20, 1090 Vienna, Austria

*[maria.frey@muv.ac.at](mailto:maria.frey@muv.ac.at)

Supplemental Material

Raw data for Figure 1

| Age | New-onset AF after SP-ablation | AF general population (Rotterdam Registry) |
| --- | --- | --- |
| 30 - 35 | 10.0 |  |
| 35 - 40 | 7.7 |  |
| 40 - 45 | 0.0 |  |
| 45 - 50 | 5.3 |  |
| 50 - 55 | 10.3 |  |
| 55 - 60 | 3.1 | 0.7 |
| 60 - 65 | 7.3 | 1.7 |
| 65 - 70 | 20.8 | 4.0 |
| 70 - 75 | 13.3 | 6.0 |
| 75 - 80 | 25.9 | 9.0 |
| 80 - 85 | 4.3 | 13.5 |
| >85 | 33.3 | 17.8 |
